# In Silico Prediction of the Mechanism of Action of Pyriproxyfen and 4′-OH-Pyriproxyfen against *A. mellifera* and *H. sapiens* Receptors

**DOI:** 10.3390/ijms22147751

**Published:** 2021-07-20

**Authors:** Giulia Spaggiari, Nadia Iovine, Pietro Cozzini

**Affiliations:** Molecular Modeling Lab, Department of Food and Drug, University of Parma, Parco Area Delle Scienze 17/A, I-43124 Parma, Italy; giulia.spaggiari@unipr.it (G.S.); nadiaiovine@gmail.com (N.I.)

**Keywords:** molecular dynamic simulations, computational methods, nuclear receptors, bees, endocrine disruptors compounds, pesticides

## Abstract

Background. Poisoning from pesticides can be extremely hazardous for non-invasive species, such as bees, and humans causing nearly 300,000 deaths worldwide every year. Several pesticides are recognized as endocrine disruptors compounds that alter the production of the normal hormones mainly by acting through their interaction with nuclear receptors (NRs). Among the insecticides, one of the most used is pyriproxyfen. As analogous to the juvenile hormone, the pyriproxyfen acts in the bee’s larval growth and creates malformations at the adult organism level. Methods. This work aims to investigate the possible negative effects of pyriproxyfen and its metabolite, the 4′-OH-pyriproxyfen, on human and bee health. We particularly investigated the mechanism of binding of pyriproxyfen and its metabolite with ultraspiracle protein/ecdysone receptor (USP-EcR) dimer of *A. mellifera* and the relative heterodimer farnesoid X receptor/retinoid X receptor alpha (FXR-RXRα) of *H. sapiens* using molecular dynamic simulations. Results. The results revealed that pyriproxyfen and its metabolite, the 4′-OH- pyriproxyfen, stabilize each dimer and resulted in stronger binders than the natural ligands. Conclusion. We demonstrated the endocrine interference of two pesticides and explained their possible mechanism of action. Furthermore, in vitro studies should be carried out to evaluate the biological effects of pyriproxyfen and its metabolite.

## 1. Introduction

*Apis mellifera* is the most widespread species in Europe among the Apis genus. Being pollinator insects, bees play a fundamental role in the environment by promoting pollination that makes them important, if not necessary, for many crops and for the maintenance of biodiversity [1]. The Food and Agriculture Organization of the United Nations (FAO) estimates that of the 100 crop species that provide 90% of food worldwide, 71 are pollinated by bees (EFSA, The European Food Safety Authority). Since 1962, bees are used as bioindicators for environmental pollution in a two-fold way: (i) monitoring the mortality; (ii) monitoring the presence of pollution residues in honey, pollen, and bee larvae [2]. In the last 10–15 years, bees’ mortality and colony losses have increased [3,4,5]. The cause for this increase is a combination of factors that affect bees vitality: virus, pathogen, invasive species, and the increasing use of pesticides. The high use of pesticides, first introduced in 1960, causes their persistence in air, soil, and water [6]. Pesticides are substances or mixtures of substances that are mainly used in agriculture to protect plants from weeds (herbicides), fungus (fungicides), insects (insecticides), and rodents. In fact, some compounds are degraded by light, soil bacteria, or chemical processes, while other compounds persist in air, soil, and water [7]. This causes the constant exposure of living beings to many substances that can have harmful effects. Agriculture is the largest consumer but pesticides are also used in public health activities to control vector-borne diseases, unwanted plants and to suppress the proliferation of insects, bacteria, and others [6,7]. However, exposure to pesticides can be extremely hazardous to humans and other non-invasive species, such as bees, causing 300,000 deaths worldwide every year [8,9]. Pesticides can cause acute health effects (such as stinging eyes, rashes, blisters, blindness, nausea) or chronic adverse effects (such as cancers, birth defects, reproductive harm, neurological and developmental toxicity, immunotoxicity, and disruption of the endocrine system) that can occur months or years after exposure [6,10,11]. Some people, such as infants and young children, are more vulnerable than others to pesticide impacts [12]. In 2002, the World Health Organization (WHO) recognized several pesticides as endocrine disruptors compounds (EDCs), which act on the endocrine system, causing adverse health effects in different organisms and their offspring [12,13]. Endocrine disruptors can act, mimic, or partially mimic the natural hormones in the body altering their metabolism [14]. Many of the insects’ endocrine systems are used as targets for the synthesis of pesticides (46% are insecticides, 21% herbicides, and 31% fungicides) that act as endocrine disruptors [15].

Pyriproxyfen, defined as an insect growth regulator (IGR), is the active ingredient used since 1995 in several insecticides, both as a single compound and in combination with other compounds [14,16,17]. EFSA declared that pyriproxyfen cannot be considered an endocrine disruptor for mammals because there are not sufficiently toxicological studies where adverse effects were observed; while in the case of bees, it stated that the proofs indicated a high risk for the larvae [17,18]. Pyriproxyfen acting as a juvenile hormone analogue (JHA) blocks the development of larvae and thus increases mortality, while sub-lethal doses affect the behavior of bees and create malformations at the adult organism level [19,20,21]. These malformations cause problems in the behavior and recognition of bees by the colony [22]. This failure to recognize both larvae and adult has the final effect of an increase in mortality, as it affects the stability and growth of the colony [19,21]. Pesticides can undergo chemical change after contact with light, heat, soil, plant and after ingestion by an animal with lower or higher toxicity than the pesticide. More than ten metabolites of pyriproxyfen have been characterized in soil, water, plants, mammals, and insects. One of the main metabolite is 4′-OH-pyriproxyfen (4′-OH-PPF) that is generated by the degradation of pyriproxyfen in soil, but also rats and mice [21,23,24,25,26].

As a juvenile hormone analogue, pyriproxyfen can affect the function of the ecdysone receptor interacting with the ultraspiracle protein (USP). Ultraspiracle protein/ecdysone receptor (USP-EcR) dimer is an arthropod receptor and is composed of two monomers: EcR (NR1H1) and USP (NR2B4), the latter is an ortholog of RXR (retinoid X receptor, NR2B1), the receptor for the vitamin A metabolite 9-cis-retinoic acid (9-cis-RA) [27,28,29]. 20-hydroxyecdysone (20E) has been identified as the natural ligand of EcR, on the contrary, the natural ligand of USP has not yet been identified even if several studies have highlighted the possible binding of USP with juvenile hormones (JHs) [29,30,31]. Henrich and co-workers studied the possible similarity of EcR to the human FXR (farnesoid X receptor, NR1H4) [32]. Farnesoid X receptor is a member of nuclear receptor family that is highly expressed in the liver, intestine, kidney, and adrenal glands, and is involved in maintaining many metabolic pathways, such as bile acid regulation, cholesterol metabolism, glucose and lipid homeostasis [33]. To activate the expression of its target genes, FXR heterodimerizes with another nuclear receptor, the retinoid X receptor α (RXRα). The alteration of expression and function of this heterodimer has been reported as a contributing factor in the development of many cancers and other diseases, such as insulin resistance, liver cirrhosis, cholestasis, coronary and crohn diseases, liver and cardiovascular diseases [34].

Given the lack of information on the risks from pyriproxyfen and its metabolites in animals and in humans, we investigated their possible negative effects on the human and bees health applying molecular modeling techniques. We particularly investigated the mechanism of binding of pyriproxyfen and its metabolite, 4′-OH-pyriproxyfen, against USP-EcR bees dimer and the relative human heterodimer RXRα-FXR. Once the USP and the EcR models were built and the interactions in the USP-EcR and RXRα-FXR dimers interface were studied, molecular docking has been carried out, in order to predict and evaluate the structural physical interactions between the receptor and the pesticides. We thought that a computational study based on nanosecond time-scale molecular dynamic simulation constitutes an appropriate approach to analyze the dynamic behavior of receptors of the bees and humans, USP-EcR and RXRα-FXR, respectively. Moreover, we analyzed the interactions with the natural ligand and with pyriproxyfen and its metabolite.

## 2. Results and Discussion

### 2.1. Molecular Model of USP and EcR

**Figure 1:**
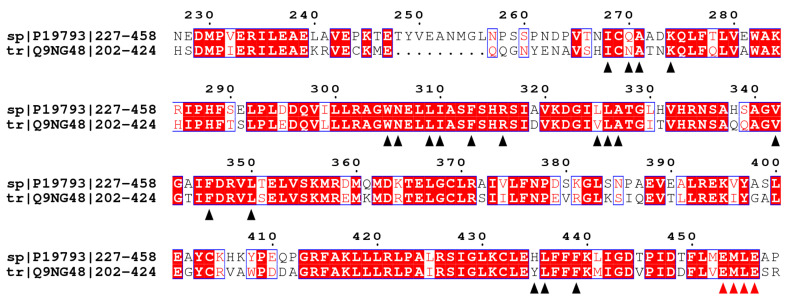
The proteins sequence alignment of *H. sapiens* RXRα (sp|P19793|) and *A. mellifera* USP (tr|Q9NG48|). The alignment was done using ClustalX and ESPript. Black arrows indicate that the residues in *H. sapiens* bind the natural ligand. Red arrows indicate the EMLE sequence important for the stabilization of H12 in *H. sapiens*. The numbering at the top refers to the sequence of *H. sapiens*.

The structure of EcR was obtained using the two monomers modeled with a homology model approach [35]. Before monomers construction, the similarity between the sequences was verified using the sequences of the LBD present in UniProt (https://www.uniprot.org/. Archived on 10 October 2019): Q9NG48 for USP, and A2PZF8 for EcR. The analysis of the sequences showed that USP has an identity of 69.51% with the RXRα of *H. sapiens* [36]. Using the Clustal (https://www.ebi.ac.uk/Tools/msa/clustalo/. Archived on 22 October 2019) program, comparison of the two sequences was done to verify the preservation of residues important for the ligand binding and for the stabilization of the H12 in an agonist conformation. These residues were identified through literature research (Ala271, Ala272, Gln275, Trp305, Asn306, Phe313, Arg316, and Cys432) [29]. As shown in the alignment (Figure 1) most of the residues are preserved. There are exceptions for some residues that are replaced by residues with the same chemical properties, Gln270 is replaced by Asn236, Leu315 is replaced by Val291. The only exception is His435 that is replaced in USP by Tyr401. This could affect the ligand binding. Moreover, the structures of USP and RXRα were compared in the binding pocket region to analyze the preservation of the residues and of the structure.

**Figure 2:**
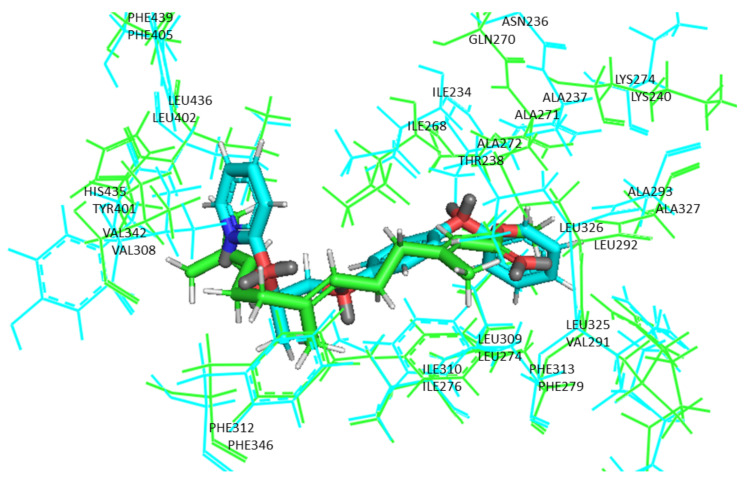
The binding pocket residues of RXRα (in blue) and USP (in green) in complex with pyriproxyfen and juvenile hormone III, respectively.

The binding pocket residues found in the RXRα structure and those found in the USP model of *A. mellifera* are preserved in both the sequence and the structure (Figure 2).

In addition, through the overlapping of the structures, it was also possible to see how the width of the binding pocket does not change. The sequence of FXR has an identity of 36% with the EcR of *A. mellifera*. Because this value is pretty low, we searched for a better sequence for the modeling using BLAST. We used the bee’s EcR LBD sequence as a query and setting an expected threshold of 10-5 in order to obtain sequences with a high identity for the model construction (identity values greater than 40%). Among the results obtained with BLAST, only sequences with a PDB crystallographic structure were taken into account: USP of *Heliothis virescens* (PDB ID: 2R40), USP of *Brucella ovis* (PDB ID: 4OZT), and USP of *Tribolium castaneum* (PDB ID: 2NXX). These sequences were aligned to analyze the sequence identity and after comparing the different sequences with the bee’s sequence, given the greater similarity, the EcR of *T. castaneum*, which has an identity of 85.90% to EcR LBD of *A. mellifera*, was used as a template. The two monomers were modeled using the LBD sequences of Apis mellifera and two different templates, whose structure was taken from PDB: RXRα of *H. sapiens* (PDB ID: 1FM6) for modeling of USP and EcR of *T. castaneum* (PDB ID: 2NXX) for modeling of EcR. The homology modeling was carried out using four software in order to obtain four structures to compare using the z-score and the G-factor values to evaluate their stereochemistry and energy distribution. Using this method is possible to obtain a more reliable structure. The structure modelled with Chimera-MODELLER was used for USP (z-score: −6.7; G-factor: −0.12) and the one modelled with Phyre2 was used for EcR (z-score: −8.27; G-factor: 0.34).

### 2.2. Comparison of A. mellifera and H. sapiens Models

**Figure 3:**
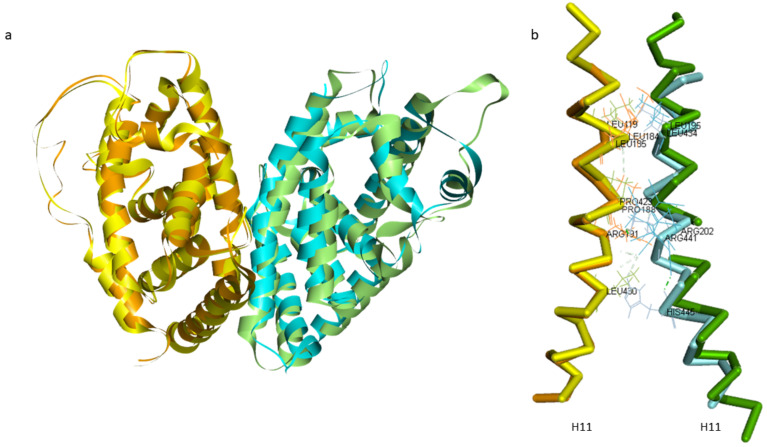
RXRα-FXR and USP-EcR dimers. (**a**) Alignment of the dimer of *A. mellifera*, USP (yellow) and EcR (green), and *H. sapiens*, RXRα (orange) and FXR (cyan). (**b**) Focus on the intermolecular interactions mediated by helices 11. Key residues that form the core hydrophobic interface of the parallel coiled are labelled.

Two monomers of *A. mellifera*, USP and EcR, and *H. sapiens*, RXRα (PDB ID: 1FM6) and FXR (PDB ID: 4QE6), were superimposed on the RXRα-FXR dimer structure of *H. sapiens* (PDB ID: 6A5Y) in order to place them at the right distance for the formation of the dimer. Through literature research, information was obtained on the interface surface of the two bee monomers and on the presence of amino acids important in the interaction of monomers for the formation of the dimer in some insects *B. ovis*, *T. castaneum*, and *H. virescens* [37]. The structural similarities of the EcR-USP of *A. mellifera* and RXRα-FXR of *H. sapiens* were analyzed, and specifically the interactions between the two monomers, to assess whether there is preservation in the interaction for the formation of the dimer. The intermolecular interactions are grouped into polar and nonpolar interactions. The nonpolar interactions include Van der Waals’s contacts and hydrophobic interactions with a distance cutoff of 4.5 Å, while the polar interactions include charged interactions (5.5 Å cutoff) and hydrogen bonds (4.0 Å cutoff). Close examination of the two dimers interfaces reveals that the residues present in the interface surfaces and residues that promote the interaction between monomers are preserved except for one residue: Asn206 in the EcR structure is replaced by His445 in the FXR structure. The interactions between both USP and EcR and RXRα and FXR were stabilized by a combination of hydrophobic and electrostatic interactions of the monomers: (i) in *A. mellifera* the nitrogen atom of Arg191 in USP makes an electrostatic interaction with the oxygen atom in EcR, while Pro188, Leu184, and Leu185 in USP form hydrophobic interactions respectively with Arg202 and Leu195 in EcR; (ii) in *H. sapiens* Leu419, Pro423, and Leu430 in RXRα make hydrophobic interactions respectively with Leu434, Arg441, and His445 in FXR (Figure 3) [33,38]. RXRα and FXR interact via the conserved asymmetric dimer interface composed mainly of H11 in each monomer [39]. Comparing the structures, the interface among all dimers is very similar, both in terms of the distance between monomers and in the secondary structures involved in the interaction between them.

### 2.3. Molecular Docking

Molecular docking was carried out using two software, GOLD (http://www.ccd.cam.ac.uk. Archived on 28 November 2019) and AutoDock [40]. The purpose of using more software is to have the possibility to compare multiple scoring functions, which have different protein–ligand interaction assessment methods. This allowed us to have more reliable results because, if three scoring functions provide a positive result, the prediction turns out to be more valid. The combination of different scoring functions allows to reduce the number of false positive, leading to more reliable results. It has been previously highlighted how the combination of three different scoring functions enhances the capability to reach hit rates from 10% up to 65–70% [41,42]. In particular, the GOLD software was used to generate the poses of each chemical, which were scored with Gold Score, Chem Score, and Hint Score, while the AutoDock software was used to generate and to score the poses with the internal scoring function. For each chemical four scoring values were obtained and their binding affinities were scored in comparison to the respective natural ligand, used as a reference compound. The use of multiple scoring functions allowed the comparison of the results obtained and the calculation of a consensus score. The natural ligands chosen were: 9-cis-retinoic acid for RXRα, chenodeoxycholic acid (JN3) for FXR, juvenile hormone III (JHIII) for USP, and 20-hydroxyecdysone for EcR [29,30,31,43,44,45]. Docking results of the ligands are illustrated in Table 1.

**Figure 4:**
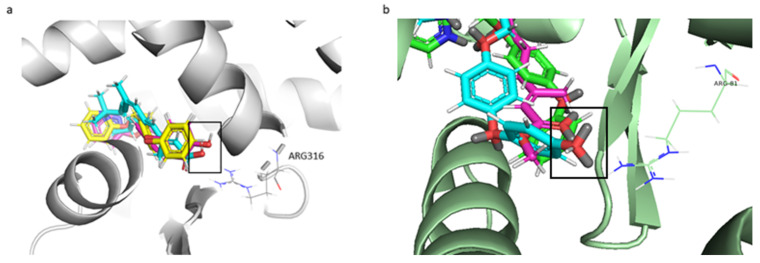
Ligand-receptor interactions. (**a**) The human hydrogen bond formed between the oxygen of 9-cis-RA (cyan) and 4′-OH-PPF (magenta) and Arg316. The box highlights the oxygen present in 9-cis-RA and the 4′-OH-PPF, but not in the pyriproxyfen (yellow). (**b**) The bee hydrogen bond formed between the oxygens of the juvenile hormone (cyan) and 4′-OH-PPF (magenta) and Arg81. The box highlights the oxygen present in the juvenile hormone and 4′-OH-PPF. The pyriproxyfen (green) does not have the oxygen for the hydrogen bond.

The approach of these software is to dock/score thoroughly all possible positions of each ligand in the binding site. The docking of the molecules was successful as indicated by the statistically significant scores, except for the Hint Score of 20-hydroxyecdysone. 20E was considered the natural ligand of EcR. It is known that the natural ligand, by definition, interacts and binds with the protein. Analyses were made to assess if the problem was the modeled structure of EcR. A new Hint Score calculation was carried out using the crystal structure of the ligand-binding domains of the *T. castaneum* heterodimer EcR-USP (PDB ID: 2NXX) bound to Ponasterone A (P1A) obtaining a negative result. We decided not to consider the Hint Score for the docking of the EcR monomer because we achieved a negative Hint Score using both natural ligand (20E) and ligand inside the pocket of the crystallized structure (P1A). Moreover, by using multiple scoring functions to obtain a consensus and having three scoring functions out of four that predicted a positive interaction, probably HINT cannot reliably predict possible interaction in the case of the EcR monomer. As shown in Table 1 the molecular docking values indicated that the binding of pyriproxyfen and 4′-OH-PPF with RXRα and USP monomers is stronger than that of 9-cis-RA and JHIII, respectively. As shown in Figure 4a, 4′-OH-PPF interacts through the same binding mode of 9-cis-RA: the formation of a hydrogen bond between the oxygen of the two ligands and active site residue Arg316 [46,47,48]. In the case of pyriproxyfen, the hydrogen bond is not present, but there are many small hydrophobic interactions with residues in RXRα: Ala272 and Val349.

Moreover, in bees as we discovered for human, the ligand interacts with an arginine residue present in the USP active site. Arg81 of the *A. mellifera* is in the same position as Arg316 of RXRα which, as said before, makes important interaction for the ligand binding. As shown in Figure 4b the interaction is a hydrogen bond between the Arg81 and the oxygens of the 4′-OH-PPF and the juvenile hormone. Pyriproxyfen does not have this interaction because of the presence of a benzenic ring in the position instead of oxygen. This benzenic ring does not interact with the arginine but makes interactions with the Ala36 as in humans. Among all the interactions some are present between residues important for the ligand binding, like Ile33 and Val107, and all three ligands.

### 2.4. Molecular Dynamic Simulations

**Figure 5:**
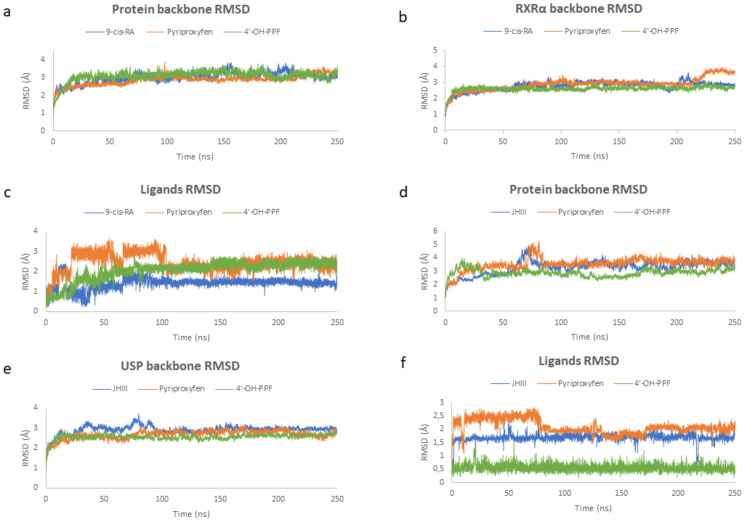
RMSD results graphics in *Homo sapiens* and *Apis mellifera*. RMSD of protein backbone of *H. sapiens* (**a**) and *A. mellifera* (**d**), RMSD of RXRα (**b**) and USP (**e**) monomer, and heavy atoms of the ligands of *H. sapiens* (**c**) and *A. mellifera* (**f**).

To evaluate the stability and the mechanism of interaction of pyriproxyfen and 4′-OH-PPF with *A. mellifera* and *H. sapiens*’ dimers, 250 ns of molecular dynamic (MD) simulations were carried out for six different complexes: (i) RXRα-FXR with 9-cis-RA and JN3, respectively; (ii) RXRα-FXR with pyriproxyfen and JN3, respectively; (iii) RXRα-FXR with 4′-OH-PPF and JN3, respectively; (iv) USP-EcR with JHIII and 20E, respectively; (v) USP-EcR with pyriproxyfen and 20E, respectively; vi) USP-EcR with 4′-OH-PPF and 20E, respectively. The root-main-square-deviation (RMSD) of the protein backbone was used to monitor conformational changes and, hence, the stability of each system during the total simulation run. From Figure 5a, it can be seen that the RMSD value of the protein backbone (RXRα-FXR) for the three *H. sapiens* systems increased ranging from 1.0–3.5 Å and ultimately attained equilibrium at about 50 ns. Upon binding, the averaged RMSD for the complex of RXRα-FXR with 9-cis-RA, pyriproxyfen, and 4′-OH-PPF was 3.04, 2.95, and 3.14 Å, respectively. As we can see from the graph, the dimer in complex with 9-cis-RA and with 4′-OH-PPF have the same constant and stable trend. To get insights into the stability of the systems, the RMSD value of the RXRα monomer backbone was calculated. From Figure 5b, it can be seen that RXRα is more stable when in complex with 4′-OH-PPF (RMSD average 2.64 Å) than when in complex with pyriproxyfen (RMSD average 3.01 Å) and natural ligand (RMSD average 2.81 Å). This is probably due to the fact that 4′-OH-PPF establishes with a protein residue a hydrogen bond reducing the conformational flexibility of RXRα compared to the pyriproxyfen. Thus, the RMSD of each ligand, 9-cis-RA, pyriproxyfen, and 4′-OH-PPF, with respect to the initial positions of the ligand atoms was evaluated for each complex. Figure 5c shows the RMSD plot of 9-cis-RA, pyriproxyfen, and 4′-OH-PPF molecules present in the active site of RXRα. During the simulation, after 40 ns, there is no significant fluctuation in the 9-cis-RA and 4′-OH-PPF molecules when they are present in RXRα; the corresponding maximum RMSD value is 2.4 Å and 2.9 Å, respectively. The stability of these two molecules in the binding cavity is due to the hydrogen bonds that limit the fluctuations of the protein–ligand complexes. Contrary to these two molecules, in the pyriproxyfen-RXRα complex, this trend is found to be different, wherein the RMSD of the pyriproxyfen molecule is relatively greater when compared with the other two systems.

In the case of *A. mellifera*, the RMSD value of the protein backbone (USP-EcR) for the two systems (the dimer in complex with juvenile hormone and the dimer in complex with pyriproxyfen) increased ranging from 1.0–5 Å and ultimately attained equilibrium at about 80 ns, while for the dimer in complex with 4′-OH-PPF—increased ranging from 1.0–3.5 Å and ultimately attained equilibrium at about 40 ns (Figure 5d). Upon binding, the averaged RMSD for the complex of USP-EcR with JHIII, pyriproxyfen, and 4′-OH-PPF was 3.29, 3.56, and 2.87 Å, respectively. As we can see from the graph, the dimer in complex with 4′-OH-PPF has the same constant and stable trend for the molecular dynamic simulation. To get insights into the stability of the systems, the RMSD value of the RXRα monomer backbone was calculated. The Figure 5e shows that USP is more stable with the 4′-OH-PPF (RMSD average 2.55 Å) and with pyriproxyfen (RMSD average 2.66 Å) than natural ligand (RMSD average 2.91 Å). Thus, the RMSD of each ligand, juvenile hormone, pyriproxyfen, and 4′-OH-PPF, with respect to the initial positions of the ligand atoms was evaluated for each complex (Figure 5f). During the simulation, there is no significant fluctuation in the 4′-OH-PPF molecules when they are present in USP. The stability of this molecule in the binding cavity is due to the hydrogen bonds that contribute to the small fluctuations of the protein–ligand complex. A similar trend is seen in the pyriproxyfen graph where at about 80 ns there is an RMSD decrease and it ultimately attains equilibrium.

The results obtained for *H. sapiens* and *A. mellifera* show that in both cases the pyriproxyfen and the 4′-OH-pyriproxyfen are stable during the dynamic simulations, and there is no difference between the dimer in complex with the natural ligands and the dimer in complex with the pesticides. One difference, in both cases, is that the dimer in complex with the 4′-OH-pyriproxyfen results to be more stable with respect to the natural ligands and the pyriproxyfen and the ligands have no significant fluctuation due to the hydrogen bonds presents in both cases.

**Figure 6:**
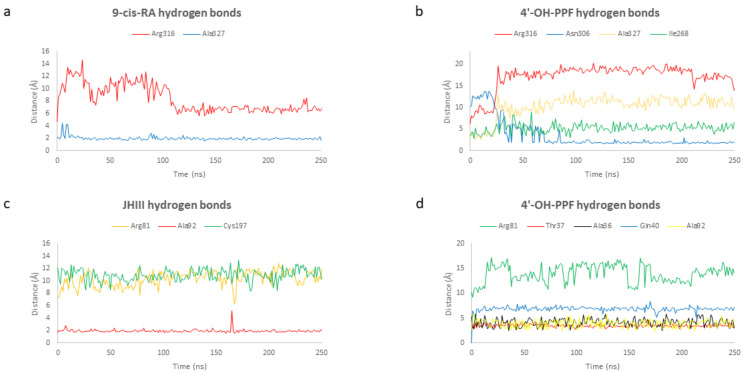
Distances between 9-cis-retinoic acid (**a**) and 4′-OH-pyriproxyfen (**b**) and the residues of RXRα involved in the hydrogen bond interactions with the ligands. Distances between juvenile hormone (**c**) and 4′-OH-pyriproxyfen (**d**) and the residues of USP involved in the hydrogen bond interactions with the ligands.

Comparing the formation and persistence of hydrogen bonds network between the ligands and the protein, it is worth to note that the interaction between 9-cis-retinoic acid and RXRα, and 4′-OH-pyriproxyfen and RXRα is characterized by the formation and breaking of two hydrogen bonds: one occurs only for few nanoseconds at the beginning of the simulation and the second one is maintained during the total simulation. In fact, the residues involved in the ligand-binding during the simulation are equal for the two complexes. Contrarywise, pyriproxyfen establishes with residues of RXRα only weak and small hydrophobic interactions resulting in a major instability of the system. The interaction between the oxygen both 9-cis-RA and 4′-OH-PPF and Arg316 breaks at the beginning of the simulation, while the hydrogen bond between 9-cis-RA and 4′-OH-PPF and Ala327 and Asn306 is mostly stable for the total simulation run, contributing to the 42.50% and 49.58% followed by the interaction with Ile268 (1.25%) and Ala327 (2.08%) respectively. These interactions are explained by the graphs showing the distances between the residues of the protein and 9-cis-retinoic acid and 4′-OH-pyriproxyfen (Figure 6a,b). The same situation of RXRα is detected for USP. As we can see in Figure 6c,d the interaction between the oxygen of both the juvenile hormone and 4′-OH-PPF and Arg81 breaks at the beginning of the simulation. After that, JHIII and 4′-OH-PPF form other hydrogen bonds with some residues in the binding cavity: Ala92 (53.00%) and Cys197 (0.04%) in the case of juvenile hormone and Ala92 (48.28%), Thr37 (0.12%), Ala36 (0.04%), and Gln40 (0.22%) in the case of 4′-OH-pyriproxyfen.

**Figure 7:**
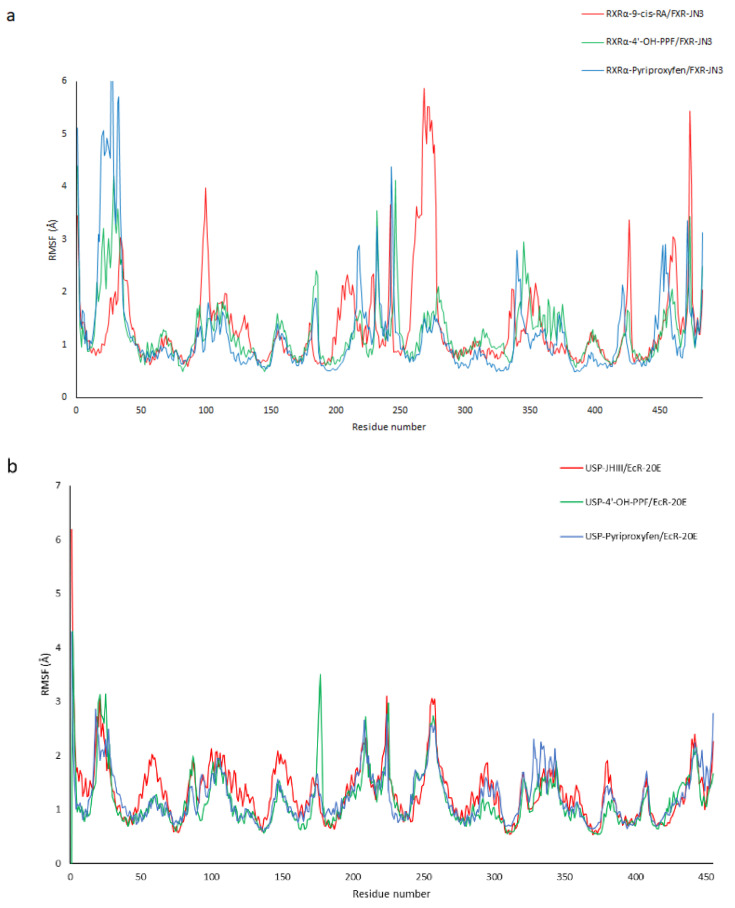
RMSF of the three *H. sapiens* (**a**) complexes and the three *A. mellifera* complexes (**b**) obtained by molecular dynamic simulations.

In addition, the root main square fluctuation (RMSF) of the six complexes was monitored to analyze the local mobility of protein residues. As shown in Figure 7, the three *H. sapiens* (a) complexes and the three *A. mellifera* (b) complexes had a similar trend.

**Figure 8:**
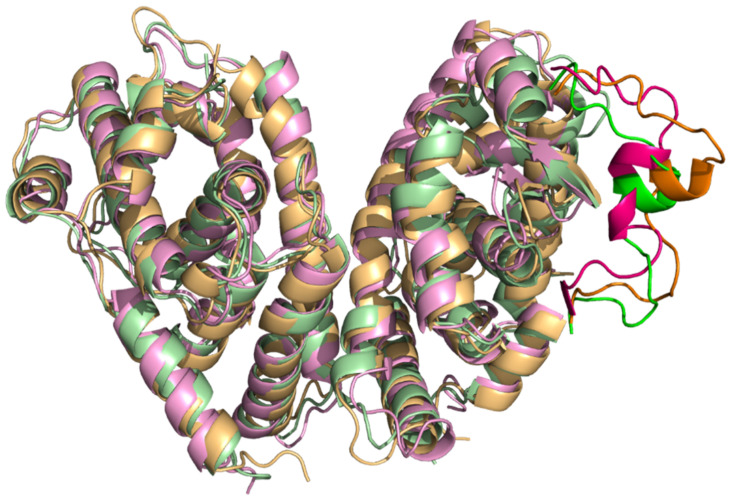
Alignment of RXRα-FXR in complex with 9-cis-RA and JN3 respectively at 0 ns, 125 ns, and 250 ns. Only relevant conformational changes have been highlighted.

However, in the case of RXRα-FXR in the regions corresponding to the amino acids from 324 to 330 and from 453 to 460, greater fluctuations were evident in the 9-cis-RA complex compared to the other two systems (Figure 8). This is due probably to the major instability of the RXRα-9-cis-RA system.

## 3. Materials and Methods

### 3.1. Molecular Model of USP and EcR

Since the structure of USP and EcR monomers of *A. mellifera* is not available in Protein Data Bank (PDB), they were modeled using homology modeling techniques. Homology research was carried out using BLAST (National Center for Biotechnology Information, 8600 Rockville PikeBethesda MD, 20894 USA) setting Refseq as a database, an Expected Threshold of 10^−5^, and a max target of 1000 [49]. The sequences of USP (UniProt: Q9NG48) and EcR (UniProt: A2PZF8) of *A. mellifera* used as query sequences for the homology research were found in UniProt [50]. The two monomers were modeled using the LBD sequences and two different templates: *H. sapiens* RXRα (PDB ID: 1FM6) for the modeling of USP and *T. castaneum* EcR (PDB ID: 2NXX) for the modeling of EcR. In order to obtain different structures to be compared four software were used for the modeling: SWISS-MODEL (Protein Structure Bioinformatics Group c/o Prof. Torsten Schwede Swiss Institute of Bioinformatics Biozentrum, University of Basel Klingelbergstrasse 50/70 CH-4056 Basel/Switzerland), I-TASSER (Iterative Threading ASSEmbly Refinement, 100 Washtenaw Avenue, Ann Arbor, MI 48109-2218), Phyre2 (Protein Homology/analogY Recognition Engine V 2.0), and Chimera MODELLER (UCSF RBVI) [51,52,53,54,55,56,57,58,59]. The reliability of the models was checked using ProSA-web (Protein Structure Analysis) and Procheck (EMBL-EBI, Wellcome Trust Genome Campus, Hinxton, Cambridgeshire, CB10 1SD, UK) which provide, respectively, z-score and G-factor values, in order to evaluate their stereochemistry and energy distribution [60,61,62,63,64].

### 3.2. Preparation of Proteins

The crystal structures of human RXRα (PDB ID: 1FM6) and FXR (PDB ID: 4QE6) monomers were downloaded from the Protein Data Bank (PDB). Both the crystallographic structure and the predicted structural models of USP and EcR monomers were processed using Sybyl software v8.1 (www.tripos.com. Archived on 5 November 2019): water molecules and ligands were removed, hydrogen atoms were added, and energy was minimized using the Powell algorithm with a coverage gradient of ≤0.5 kcal (mol Å)−1 and a maximum of 1500 cycles. However, for the docking with AutoDock (see below), the receptors were further processed as follows: the AutoDockTools software was used to add polar hydrogen to the proteins and the Gasteiger charges were calculated for each atom and to assign AD4 type to the atoms.

### 3.3. Preparation of Ligands

The structural coordinates of the ligands, such as juvenile hormone III, 9-cis-retinoic acid, 20-hydroxyecdysone, chenodeoxycholic acid, and pyriproxyfen were retrieved from the NCBI PubChem compound database (https://pubchem.ncbi.nlm.nih.gov/. Archived on 10 November 2019). In the case of 4′-OH-pyriproxyfen, the three-dimensional structure was built, and energy minimized with Sybyl software v8.1 using the Powell algorithm with a coverage gradient of ≤0.5 kcal/(mol Å) and a maximum of 1500 cycles. Moreover, in order to assign the correct protonation state to the ligands (pH = 7.4), the software FLAP (Fingerprint for Ligand and Protein) was used.

### 3.4. GOLD Docking

The GOLD (Protein Ligand Docking Software) software v5.8.1 (CCDC; Cambridge, UK; http://www.ccd.cam.ac.uk. Archived on 28 November 2019) was applied to dock ligands into the binding site of the receptors. For each compound and receptor, 30 binding poses were generated without any constraints. In bees cases, the centroid of the binding site was defined using the coordinates of the crystallographic complexes, 2NXX in the case of EcR monomer (#C24 of P1A: x = 29.069, y = 6.239, z = 8.576) and 1FM6 in the case of USP monomer (#C10 of 9CR: x = 17.688, y = 14.021, z = 14.525), while in the human case was used 1FM6 for RXRα (#C10 of 9CR: x = 17.688, y = 14.021, z = 14.525) and 4QE6 in the case of FXR (#C13 of JN3: x = 10.872, y = 15.018, z = 11.917). Side chain flexibility was allowed for the amino acids: EcR: Glu17, Thr52, Lys93, Phe437, Tyr114; USP: Val30, Ile33, Thr37, Lys39, Leu91, Thr93, Ile110, Leu201, Phe202; RXRα: Phe436, His435, Phe439, Leu436; FXR: Met265, Met290, His294, Phe336, Phe350, Tyr369, Met450, Trp454, Trp469. For the genetic algorithm run, a maximum number of 100,000 operations were performed on a population of 100 individuals with a selection pressure of 1.1. The number of islands was set to 5 and the niche size was set to 2. The default GOLD Score fitness function was applied for performing the energetic evaluations. The distance for hydrogen bonding was set to 2.5 Å and the cut-off value for the van der Waals calculation to 4.0 Å. For ligand flexibility options, flip pyramidal N, flip amide bonds, and flip ring corners were allowed. After that, all the poses generated by GOLD were rescored using the scoring functions Chem Score and Hint Score (HINT, Hydropathic INTeraction) with the aim to obtain a consensus.

### 3.5. AutoDock Docking

The search space was included in a box of 24 × 24 × 24 Å, centered on the binding site of the ligands as mentioned before. The side chain flexibility was allowed for the same residues defined in the GOLD docking. The ligand amide and backbone flexibility were allowed.

### 3.6. USP-EcR and RXRα-FXR Dimers and the Interfaces Key Interactions

To build the USP-EcR and RXRα-FXR dimers, the structural similarities of the structures present in PDB were analyzed using the Pymol software: 2R40 of *H. virescens*, 4OZT of *B. ovis*, 2NXX of *T. castaneum*, and 5Z12 of *H. sapiens*, in bee case, while 6A5Y (RXRα-FXR with the best resolution value) of *H. sapiens* in the human case. Discovery Studio (Dassault Systèmes BIOVIA, Discovery Studio Modeling Environment, Release 2020, San Diego: Dassault Systèmes, 2020) was used to analyze the key interactions on the interface of the two dimers.

### 3.7. Molecular Dynamic Simulations

The best molecular docking pose for each ligand–protein complex was chosen as the starting point of the molecular dynamic simulations. The protein–ligand complex was prepared using the web-based graphical user interface CHARMM-GUI (Effective Simulation Input Generator and More, Lehigh University, Bethlehem) (http://www.charmm-gui.org/. Archived on 25 January 2020). Each complex was solvated in a rectangular 15 Å water box (TIP3S). Molecular dynamic simulations were performed using the NAMD 2.13 (NAMD was developed by the Theoretical and Computational Biophysics Group in the Beckman Institute for Advanced Science and Technology at the University of Illinois at Urbana-Champaign) software package [65]. For each system, two rounds of energy minimization were performed, each comprising 0.1 ns of conjugate gradient minimization. First, a weak constraint (1.0 kcal/(mol·Å^2^)) was assigned to all heavy atoms for both the protein and the ligand, allowing the minimization of hydrogen atoms. Second, no restraints were employed in order to allow the minimization of the entire system. Both systems were gradually heated from 50 to 300 K in NVT mode (number of atoms volume temperature) for 0.2 ns, while the heavy atoms of the protein were restrained with a force constant of 0.5 kcal/(mol·Å^2^). The system was further equilibrated at constant pressure (1.0 bar) for 1 ns (NPT). Each molecular dynamic simulation was performed for 250 ns without any constraint, allowing the movement of the entire system.

## 4. Conclusions

Pesticides are widely used in agriculture worldwide. The increase of the pesticides used and their persistence in air, soil, and water is the reason why these compounds are associated with human disease and bee disease and mortality. An important nuclear receptor in bees is the ecdysone receptor that regulates the development and behavior of bees through its activation induced by hormones such as 20-hydroxyecdysone and juvenile hormone. This receptor, composed of two monomers, EcR and USP, is an ortholog of the human FXR-RXRα.

The purpose of this paper was to use in silico techniques for the prediction of endocrine interference of pyriproxyfen and its metabolite 4′-OH-pyriproxyfen on the *A. mellifera* USP- EcR dimer and the *H. sapiens* ortholog RXRα-FXR. Docking results, both for humans and bees, predicted a protein–ligand interaction for the two compounds that can be considered as possible binders for the USP and RXRα monomers. Our results show that the 4′-OH-pyriproxyfen, like the natural ligand, makes an important hydrogen bond with an Arg residue (Arg316 for humans and Arg81 for bees) that is known to be an important residue for the ligand binding. The molecular dynamic simulation allows us to analyze the stability of the dimers and of the ligands inside the binding pocket and the interactions changing and conservation during the 250 ns of simulation. Our results show how the two compounds are stable inside the binding pockets and comparing the simulation that we studied there are no significant differences between the dimers binding the two compounds and the dimers binding the natural ligand. We also showed that the 4′-OH-pyriproxyfen seems to be more stable with respect to the pyriproxyfen, both in humans and bees.

In conclusion, these in silico analyses revealed a possible interaction of the two compounds, pyriproxyfen and 4′-OH-pyriproxyfen, with RXRα-FXR and USP-EcR dimers. These interactions and possible binding to the monomers can affect the normal function of the dimers. We demonstrated the endocrine interference of these two compounds and we explained the possible mechanism of action; in vitro studies should be carried out to evaluate the biological effects of pyriproxyfen and its metabolite.

## Figures and Tables

**Table 1:** Molecular docking results of the natural ligands, pyriproxyfen, and 4′-OH-pyriproxyfen against *A. mellifera* and *H. sapiens*’ monomers.

RXRα				
Ligand	Gold Score	Chem Score	Hint Score	Affinity
9-cis-retinoic acid	72.96	39.72	1374.1	−9.9
Pyriproxyfen	59.58	35.02	1376.3	−9.8
4′-OH-pyriproxyfen	64.52	35.82	1279.4	−9.8
FXR				
Chenodeoxycholic acid	74.56	32.1	1920.2	−11.2
USP				
Juvenile hormone III	49.02	26.73	841.04	−6.9
Pyriproxyfen	60.29	32.14	1178.7	−9.0
4′-OH-pyriproxyfen	56.15	29.75	1136.0	−9
EcR				
20-hydroxyecdysone	77.58	25.98	−2580.88	−9.7

## Data Availability

Data available on request due to restrictions e.g., privacy or ethical. The data presented in this study are available on request from the corresponding author. The data are not publicly available due to the big amount of data.
